# Increasing differential diagnosis between lipoma and liposarcoma through radiomics: a narrative review

**DOI:** 10.37349/etat.2023.00147

**Published:** 2023-06-30

**Authors:** Raffaele Natella, Giulia Varriano, Maria Chiara Brunese, Marcello Zappia, Michela Bruno, Michele Gallo, Flavio Fazioli, Igino Simonetti, Vincenza Granata, Luca Brunese, Antonella Santone

**Affiliations:** University of Campania “Luigi Vanvitelli”, Italy; ^1^Department of Medicine and Health Sciences “Vincenzo Tiberio”, University of Molise, 86100 Campobasso, Italy; ^2^Orthopedics Oncology, National Cancer Institute IRCCS “Fondazione G. Pascale”, 80100 Naples, Italy; ^3^Radiology Division, National Cancer Institute IRCCS “Fondazione G. Pascale”, 80100 Naples, Italy

**Keywords:** Radiology, radiomics, soft tissue sarcomas, liposarcoma, magnetic resonance imaging

## Abstract

Soft tissue sarcomas (STSs) are rare, heterogeneous, and very often asymptomatic diseases. Their diagnosis is fundamental, as is the identification of the degree of malignancy, which may be high, medium, or low. The Italian Medical Oncology Association and European Society of Medical Oncology (ESMO) guidelines recommend magnetic resonance imaging (MRI) because the clinical examination is typically ineffective. The diagnosis of these rare diseases with artificial intelligence (AI) techniques presents reduced datasets and therefore less robust methods. However, the combination of AI techniques with radiomics may be a new angle in diagnosing rare diseases such as STSs. Results obtained are promising within the literature, not only for the performance but also for the explicability of the data. In fact, one can make tumor classification, site localization, and prediction of the risk of developing metastasis. Thanks to the synergy between computer scientists and radiologists, linking numerical features to radiological evidence with excellent performance could be a new step forward for the diagnosis of rare diseases.

## Introduction

Soft tissue tumors (STTs) are a type of mesenchymal tumor that include lipomatous tumors, fibrohistiocytic and fibrous tumors, vascular tumors, and nerve sheath tumors. The incidence of benign tumors is 3,000 cases per 1 million population and 50 cases per 1 million population for malignant ones. Age-related incidences vary but like almost all other malignancies, STTs become more common with increasing age and the median age at diagnosis is 65 years [1].

The World Health Organization (WHO) committee for the classification of STTs revised the classification in 2020 with the description of different entities dividing them into benign, intermediate (locally aggressive), and malignant lesions (Table 1) [2]. Since these entities have similar radiological features, they can be challenging from the diagnostic point of view.

**Table 1:** Lipomatous tumors WHO classification of STTs 2020

**Type**	**Classification of lipomatous tumors**
Benign	Lipoma, lipomatosis, lipomatosis of nerve, lipoblastoma and lipoblastomatosis, angiolipoma, myolipoma of soft tissue, chondroid lipoma, spindle cell lipoma and pleomorphic lipoma, hibernoma, atypical spindle cell/pleomorphic lipomatous tumor
Intermediate	ALT/WD-LPS
Malignant	DD-LPS, M-LPS, PM-LPS, MPM-LPS

ALT: atypical lipomatous tumor; WD-LPS: well-differentiated liposarcoma; DD-LPS: dedifferentiated liposarcoma; M-LPS: myxoid liposarcoma; PM-LPS: pleomorphic liposarcoma; MPM-LPS: myxoid pleomorphic liposarcoma

The degree of malignancy refers to the classification by the National Federation of Cancer Centers (French, FNCLCC) recommended by the WHO: (A) grade 1 (G1): low-G; (B) G2: intermediate-G; (C) G3: high-G [3–11].

Lipomatous tumors are the second most common subgroup of STTs in adults, with a peak age of incidence between the fifth and seventh decade [11, 12]. Considering the rarity of malignant tumors and the frequency of the benign ones, malignant lesions are often inadvertently presumed to represent benign lesions, and unproper excisions increase patient morbidity [4].

In this context, the aim of this narrative review is to report the main radiological characteristic of this sub-type of tumors, to increase the knowledge for beginners and residents. As shown in Table 1, the WHO organization provided 2020 a classification of STTs [2].

## Lipoma and LPSs features

Lipoma and other benign adipocytic tumors are the most common STTs with at least 30% of the benign tumors of soft tissue being lipomas [4]. There are not precise data about the incidence; as these tumors are often underreported because many are asymptomatic, and medical attention is not obtained. Local recurrence of lipomas after marginal excision is 5%, but it may be more familiar with infiltrating intramuscular lipomas [13, 14].

LPSs are intermediate and malignant lipomatous STTs accounting for approximately 20% of all soft tissue sarcomas (STSs) [1] and have a significant potential for metastasis and recurrence [15]; on the contrary, lipomas do not present a risk of local progression and metastasis.

The intermediate (locally aggressive) tumors are ALT/WD-LPS; the malignant ones are M-LPS, DD-LPS, PM-LPS, and MPM-LPS [12, 16].

ALTs and WD-LPS most commonly occur in middle-aged adults with a peak incidence in the sixth and seventh decades. This lesion may best be conceptualized as precancerous, and the risk of malignant transformation depends on the anatomic location and lesion duration. The term ALT is used for extremity-based or superficial trunk lesions preferring the term WD-LPS for masses arising from the retroperitoneum, the mediastinum, or para testicular locations [4]. These clarifications have surgical implications and different recurrence risks with the high rate of WD-LPS. ALT/WD-LPS reported local recurrence rates to vary from 8.2% to 50.61%, with the most extensive series of 151 patients demonstrating a 10% risk. Local recurrence occurs on average 6 years to 8 years after surgical excision. Lesions that do recur may have a higher risk of recurrence, reported at 52%, and the risk of malignant dedifferentiation ranges from 0% to 5%. Complete surgical excision of ALT/WD-LPS is recommended to decrease the risk of local recurrence [17–19].

DD-LPS has some morphologic and molecular in common with WD-LPS, which is because DD-LPS occurs as a focal outgrowth within precursor lesions, with 90% of DD-LPS found within a primary WD-LPS [16]. DD-LPS represents 18% of all LPSs and exhibits a high local recurrence rate, up to 100% if located in the retroperitoneum [1]. Distant metastasis occurs in 15% to 20% of cases, and 5-year overall mortality is 30%, but likely much higher at long-term follow-up [20].

Because of the similarity of ALT/WD-LPS/DD-LPS with lipoma at imaging today, genetic testing is the gold standard to distinguish lipoma from ALT/WD-LPS/DD-LPS [7].

Murine double minute 2 (MDM2) amplification on fluorescence *in situ* hybridization (FISH) studies distinguishes ALT/WD-LPS from a lipoma, with 92% to 100% specificity and 97% to 100% sensitivity. Cyclin-dependent kinase 4 (CDK4), located in the same region of chromosome 12 as MDM2, is amplified in approximately 85% to 90% of ALTs [21, 22].

**Figure 1:**
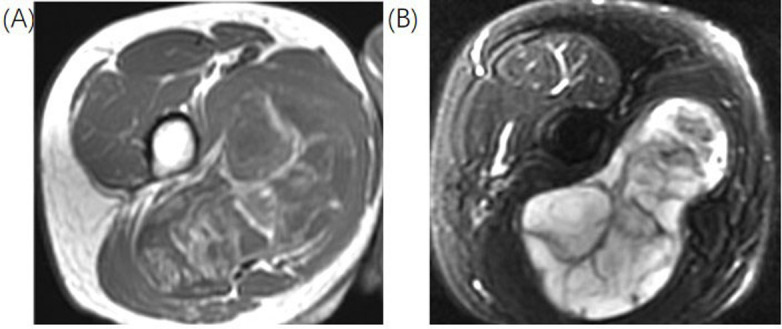
Representation of M-LPS. In this figure, there is the identification of a lesion in the medial compartment of the thigh. (A) Axial T2FS sequences with hyperintense of the lesion; (B) axial T1 sequences with hipointensity of the lesion. There are multiple internal settings of the lesion hypointense in the T2FS sequences and hyperintense in the T1 sequences. The lesion was proven at the biopsy a high-G M-LS. T2FS: T2-weighted fat-suppressed *Note.* Reprinted from Cancer Imaging Archive [Internet]. Frederick (MD): Frederick National Laboratory for Cancer Research. c2014–2020 [cited 2015 Jun 1]. Available from: https://wiki.cancerimagingarchive.net/pages/viewpage.action?pageId=21266533. CC BY.

M-LPS represents a continuum from low-G well-differentiated myxoid (WD-M) tumors, and they account for 20% of LPSs (Figure 1). The peak incidence is between the fourth and fifth decade and is the second commonest sub-type of liposarcoma (LPS) and the commonest sub-type of LPS occurring in young patients [23]. From a genetic point of view, translation (*t*) [23, 24] translocation, which leads to a FUS RNA binding protein-DNA damage inducible transcript 3 (*FUS-DDIT3*) gene fusion, is present in 95% of cases [4]. PM-LPS is the least common subtype accounting for 5% to 15% of all LPSs. Most cases occur later in life, with the peak incidence in the seventh decade with an exceptionally high rate of local recurrence and distance metastatization of 35% [25]. This tumor presents a complex genomic profile with frequent loss of RB transcriptional corepressor 1 (RB1) and alterations of protein p53 (p53). MPM-LPS is a new entity introduced in the WHO classification (2020) that shares characteristics with M-LPS and PM-LPS.

## Clinical evaluation

Clinical features are only occasionally sufficient to distinguish benign from malignant tumors of soft tissue and range from totally asymptomatic cases to pain related to the mass effect of the tumor, often with compression of the neurovascular bundle [12–16]. Typically, lipoma and other benign entities present a painless soft tissue mass with doughy consistency at palpation. This lesion can arise deep and, in that case, tend to be larger; they can arise within a muscle, classified as inter- or intra-muscular, respectively. Intramuscular lipomas will move in coordination with muscle contraction, whereas superficial lipomas will remain mobile despite muscle activation. LPSs instead present as painless enlarging soft tissue masses with a rate of growth that tends to be quicker for high-G tumors. Most patients with intra-abdominal or retroperitoneal sarcomas present with an asymptomatic abdominal mass are confirmed on abdominal imaging [13, 14]. Because there are few clinical findings and specific laboratory abnormalities, these tumors may grow large without any symptoms. When symptoms occur, they are usually nonspecific and related to mass effect or invasion of local structures.

## Imaging

Because the clinical examination is usually not helpful, the Italian Association of Medical Oncology and European Society of Medical Oncology (ESMO) guidelines suggest imaging with magnetic resonance imaging (MRI) for deep masses of any size, surface masses > 5 cm, or rapidly growing masses. They should be considered suspicious of sarcoma and treated as such or referred to the Centers of High Specialization [3, 11].

MRI allows the study of the anatomical relationships of the tumor with the adjacent structures for optimal surgical planning and guides biopsy [3–11]; therefore, the first diagnostic step is to classify the tumors as either “deep” or “superficial”, depending on the location of the investing fascia [7].

Usually, for superficial lipomas, the first diagnostic tool is ultrasound, and it will present as a homogenous hyperechogenic mass with well-defined margins [4]. If performed MRI lipomas consistently show homogenous isointense signal to fat on all sequences with homogenous low signal in the sequences with the saturation of fat signal [13]. Conversely, in the US, LPS appears as a heterogeneous, multi-lobulated, and typically well-defined mass. However, compared to lipoma, the possibility of characterizing LPS in the US is limited.

At computed tomography (CT), WD-LPS typically appears as a predominantly adipose mass containing non-lipomatous components. The non-lipomatous entities are prominent thick septa with nodularity. Focal nodular or globular non-adipose areas may also be apparent, but they are usually < 2 cm in size. Features that favor the diagnosis of WD-LPS as opposed to lipoma include male sex, age > 66 years, a lower percentage of fat, calcification presence, lesion size > 10 cm, thick septa, and non-lipomatous nodular or globular foci.

MRI allows a quality assessment of the lesion with the identification of the signs that can indicate malignancy as [7]:

(A) Nodular non-fatty areas within the mass or nodular fatty areas with different densities or signals than the subcutaneous fat;
(B) Presence of thick septa (> 2 mm) or irregular or nodular septations with enhancement after contrast injection;
(C) Significant increase in size over time by either clinical or radiological examination;
(D) Intra-tumoral calcifications;
(E) Mediastinal, retroperitoneal, intra-abdominal, or pelvic/spermatic cord origin;
(F) Most extensive length [16, 26] is over 10 cm for superficial localization and 5 cm for deep location.

Areas of active tumor enhancement on post-contrast MRI indicate areas of viable tumor that should be targeted for biopsy.

Regarding M-LPS, US appearance is characterized by a complex, well-defined, hypoechoic but solid mass with posterior acoustic enhancement. At CT, these entities have a pathognomonic appearance: typically large, well-defined, and multilobulated intermuscular lesions. The high-water content is reflected as a predominantly low density on CT images.

**Figure 2:**
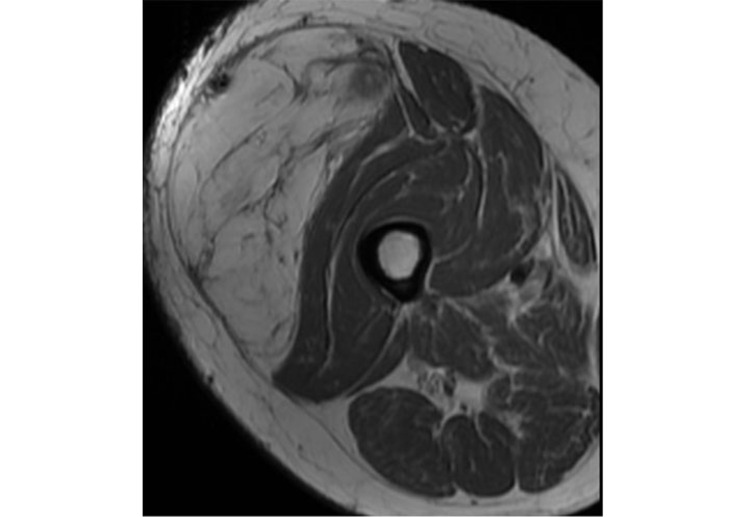
Representation of ALT/WD-LPS. In this figure, there is the identification of a large lesion of the vastus lateralis. Axial and T1 sequences with hyperintensity of the lesion and hypointense of the septa. The lesion was proven at the biopsy ALT/WD-LPS *Note.* Reprinted from Cancer Imaging Archive [Internet]. Frederick (MD): Frederick National Laboratory for Cancer Research. c2014–2020 [cited 2015 Jun 1]. Available from: https://wiki.cancerimagingarchive.net/pages/viewpage.action?pageId=21266533. CC BY.

ALT/WD-LPS usually shows as a fat-containing mass with thick irregular septa (> 2 mm) and some non-fatty areas with possible sporadic calcifications (Figure 2) [4]. The imaging findings of WD-LPS can overlap with DD-LPS, which usually larger at first diagnosis [25]. A non-lipomatous mass highlights nearby a WD-LPS is suspicious for dedifferentiation in DD-LPS. WD-LPS and DD-LPS can be multifocal at initial presentation or recurrence, often related to some underlying genetic defect [27]. DD-LPS with large non-fatty areas goes into differential diagnosis with PM-LPS.

The imaging findings of PM-LPS are typically a large, relatively well-defined, nonspecific soft tissue mass with often infiltrative margins and heterogeneous signal intensity due to the presence of hemorrhage and/or necrosis. The signal intensity of intralesional fat may be less than that of subcutaneous fat but the absence of fat within the neoplasm can make the imaging diagnosis difficult [28, 29].

M-LS findings are characteristic of an encapsulated mass with T1-hypointense and markedly T2-hyperintense with marked or heterogeneous enhancement [28]. The differential diagnosis often is an intramuscular myxoma or cystic lesion, but the marked or heterogeneous enhancement and the identification of fatty component help in the differentiation. High-G M-LPS have heterogeneous signal intensity on T1-weighted (T1W) and T2W images due to non-fatty non-myxoid (M, round cell) components mixed with M and fatty components. The non-fatty non-M (round cell) component shows intermediate T2 signal intensity and variable enhancement [30].

The conclusion of the radiological report should be written with caution. Due to overlap in imaging findings, the challenges of accurately distinguishing between lipoma and LPS must include information about anatomical location, tumor size, features of benignity or malignancy, and comorbidity.

Regarding disease stadiation, CT is the tool to choose. A CT scan of the thorax is recommended for staging purposes [7] for all kinds of malignant LPS [11, 3] with the addition of the abdomen and pelvis in M-LPS because it is common to metastasize to extrapulmonary sites. In addition, in the pre-surgical phase of WD-LPS and for the retroperitoneal location of DD-LPS or PM-LPS, CT allows the evaluation of the relationship to vital structures and assess the extent of the tumor, especially the vascular assessment [7, 11].

## Management and follow-up

Lipomas are benign entities with no risk of malignant transformation and do not require referral to an orthopedic oncologist [5] that can be managed conservatively with observation. Local recurrence of lipomas after marginal excision is 5% but may be more familiar with infiltrating intramuscular lipomas [4]. One of the great difficulties is the differential diagnosis between benign tumor (lipoma) and intermediate LPS (ALT/WD-LPS), even for the experienced pathologist on the biopsy sample [3]. The difference in the prognosis between ALT and WD-LPS may also reflect the difficulty in recognizing pathological adipose tissue in the retroperitoneum, which leads to incomplete tumor resection.

LPS is a potentially lethal disease with poor overall survival, three treatment options exist surgery, chemotherapy (CHT), and radiotherapy (RT), with surgery with wide resection and tumor-free margins, the only curative one. RT only reduces the local recurrence of an LPS, and CHT is linked to limited overall survival [31, 32]. The standard surgical procedure is an en-bloc excision with R0 margins. This implies removing the tumor in a single specimen with a rim of normal tissue around it. As an individualized option, R1 excision can be acceptable in carefully selected cases; marginal excisions along the pseudocapsule are advised for ALT [33]. RT is typically added to surgery as part of the standard treatment of high-G (G2–3) lesions [11].

WD-LPS and DD-LPS are typically radio-insensitive and chemo-sensitive in the opposite of M-LPS, which are markedly more chemo-sensitive and radio-sensitive [16]; specific to M-LPS, radiation, and CHT are particularly effective in decreasing the tumor size [34, 35].

There are few published data to indicate the optimal routine follow-up policy of surgically treated patients with localized disease, but ESMO guidelines suggest, after completion of treatment, intermediate-/high-G patients may be followed every 3–4 months in the first 2–3 years, then twice a year up to the fifth year, and once a year after that; low-G sarcoma patients may be followed every six months for the first five years, then annually [11].

## Classification with radiomics

Can radiomics help distinguish lipomas from ALT/WD-LPS? Using a database with histopathological information about the diagnosis and the MDM2 amplification, radiologists can construct a model to distinguish a lipoma from an ALT/WD-LPS using the T1W turbo spin echo (TSE) MRI series for each [24].

For the radiomic analysis, the images must be segmented choosing a region of interest (ROI) where there is the possibility to localize the disease marks. This step of segmentation can be manual or automatic: when the images are manually segmented in 2 dimensions (2D), the segmentation does not consider voxels and unfortunately, and this means it can be operator dependent. The clinical visual examination of radiologists, instead, consists to determine whether there are septae thicker than 2 mm, nodules and/or patches of non-fat tissue, and if the tumor is seated deeply or superficially.

To do image segmentation, there are different software available. From there, the feature definitions of the imaging biomarker explorer (IBEX) software are not compliant with the image biomarker standardization initiative (IBSI) standard, because its preprocessing can introduce non-negligible nonconformities with the main radiomic standard [36].

The most interesting results in the research community are those linking radiomics to clinical evidence, in particular for first-order features, the energy, and total energy values were not considered significant as lipomas have a mass that does not generate a significant negative signal. Furthermore, first-order features were not important due to their low specificity and area under the curve (AUC).

For second-order features, run length nonuniformity [belonging to gray-level run-length matrix (GLRLM) class] was the best performer, whereby a low value would indicate greater homogeneity in image lengths; gray level nonuniformity is also a performer, with a low value indicating the greater similarity of grey levels in the image. The energy of the gray-level co-occurrence matrix (GLCM) also shows higher values if there is a more chaotic texture in the image with scattering and voxels with variability.

To differentiate WD-LPSs from lipomas only concentrating on MRI scenes, it can be important to focus on the *MDM2* gene [37].

In the world of artificial intelligence (AI), it is recommended to always do multicenter studies and have at least one external validation database. In fact, these requirements ensure that the machine learning (ML) model has no problems with overfitting, underfitting, or errors and bias due to the composition of the training set.

The feature extraction is more reproducible and stable if done using pyradiomics and other literature semi-quantitative features available. From the radiological images can be extracted features of intensity, shape, and texture to include in ML models.

The introduction of the workflow for optimal radiomics classification (WORC) tool [38], includes steps of feature selection and those of ML to pattern recognition.

LPSs are larger than lipomas, so volume is a determining factor for classification. In particular, the results of T1W image performances are compared with those of T1W + T2W and those of three expert radiologists [37]. When a patient has the T2W scene included in the analysis, this can gain higher performances of classification. The performance of radiologists is significantly better in terms of sensitivity and therefore worse in specificity, while they remain similar in terms of AUC. Surely, the reliability of a medical radiologist expert can be considered better if accompanied by software of radiomics that compensates him for the disadvantaged.

The real challenge of the radiomic work is precisely to differentiate soft tissue lipoma and LPS better than musculoskeletal (MSK) radiologists [26]: this can be done by creating a predictive model with radiomics and ML that could differentiate lipomas and LPS on preoperative axial T1W MRI images and with histological information. In fact, it can be noticed in literature the robustness of the MRI T1W scene, which allows to conduct of new experiments and deepen the knowledge of these diseases.

To do a classification task using radiomics, also the clinical variables can be taken into account:

(A) Homo-/hetero-geneity;
(B) Presence or absence of thick septa;
(C) Nodular enhancement;
(D) Restriction of diffusion retailed with McNemar to compare the work of three radiologists through the kappa statistics.

Generally, the support vector machine algorithm performs well on radiomic features, first standardizing the distribution of the feature as a normal one and then applying an algorithm of dimensionality reduction; maybe this process can be repeated with several weights to differentiate the various types of LPS present in the database.

Radiomics has beaten the radiologists singularly and totally: in fact, at the best of the author’s knowledge, a radiologist arrives at 69% of accuracy. The common error lies in the atypical spindle cell present in lipomas, so MSK radiologists consider it as LPS, while the ML algorithm correctly recognizes it [26].

In literature, there are also studies that classify a certain type of LPS [39], developing and validating a model based on radiomics, MRI images, and ML for example to distinguish atypical LPS (ALT) and WD-LPS. Having the histologic information and enhanced T1W MRI from different centers, to do the radiomic analysis there is the need for a discretization of the images, which can enhance on reduce the variations in the color channel. The effect caused by discretization produced the coefficient of variation that indicates:

(A) 0–5% means the absence of variations;
(B) 5–25% means acceptable variations;
(C) 25% means nonacceptable variation.

Only 20% of the total extracted features have an acceptable variation [39]. This peculiarity should be studied more deeply to understand how much it affects the final diagnosis or the feature stability, together with the image resolution.

On one hand, LPS patients present differences between different vendors’ machines; on the other hand, the LPS patents are only different in GLCM features. This is a strong point for the interoperability of radiomics in the clinical daily utility and to assess the robustness of the method.

The provocation between the performances of radiomics and radiological experts is represented by the classification of ALT compared to LPS using the axial T1W and T2FS MRI scenes [40].

On one hand, if 3 different models are built, one for T1W features, one for T2FS features and one for T1W + T2W features, surely there are no differences in the use of T1W or T2W models for the diagnoses.

On the other hand, 3 MSK radiologists with more than 10 years of experience can do better than ML models looking at the presence of septa, the presence of nodular enhancement, and the homogeneity or heterogeneity knowing only the patient’s age as clinical information.

The results were excellent for both radiologists’ and radiomics’ models [40], but among these, there are differences; on average the AUC, specificity, and accuracy values of radiologists are lower than radiomics; only sensitivity is better in radiologists. A summary of the previous performances are displayed in Table 2. This is the confirmation that the use of radiomics can help in clinical practice and also against rare diseases, without invasive interventions or contrast enhancement imaging.

**Table 2:** Performance comparison of radiologists and radiomics

**Aim**	**Reference**	**Information database**	**Radiomic performances**
WD-LPS *vs.* lipoma	Vos et al. [37]	MDM2 detection	Sensitivity: radiologist > radiomics Specificity: radiologist < radiomics Accuracy: radiologist = radiomics
LPS *vs.* lipoma	Malinauskaite et al. [26]	Histologic examination	Sensitivity: radiologist < radiomics Specificity: radiologist < radiomics Accuracy: radiologist < radiomics
ALT *vs.* lipoma	Tang et al. [40]	Histologic examination	Sensitivity: radiologist > radiomics Specificity: radiologist < radiomics Accuracy: radiologist < radiomics

In these radiomics studies, the presence of mesenchymal cells and fat necrosis can cause nodular appearance and affect classification performances.

The research community tried to identify which visual clinical information discriminates lipoma from ALT. Maybe is possible to determine whether macroscopic fat heterogeneity can have good classification performance [41] through MRI textural analysis using a computer-aided diagnosis (CAD) tool based on radiomics [MRI textural analysis (MRTA)].

Using sagittal or coronal T1W TSE, coronal or sagittal short TI inversion recovery (STIR), and T2W fast spin echo (FSE), axial proton density-weighted (PDW) FSE and axial spectral attenuated inversion-recovery (SPAIR), a blind MSK radiologist can review the cases describing the lesions information. In particular, a coronal T1W image and a single axial PDW FSE image in digital imaging and communication in medicine (DICOM) are enough to do feature extraction. Another radiomic software is TexRad [42], in which compliance with the main standard IBSI is not known.

Radiomics realized that the presence of at least 2 variables between PDW image, location, depth, and fat content can classify an ALT with the greatest sensitivity and specificity. Furthermore, it is possible to link radiomic features with clinical evidence: of the most significant features, a negative kurtosis could indicate a lower contrast in the lesion and it can fit the gross radiological evaluation of tumors; higher mean values in lipomas imply that they are less contrasty and more uniformly hyperintense [41].

Sometimes radiomics alone is not enough, due to particular images. For this, the information can be enhanced by applying filters to the image, which allow for more contrast or blurring in the image and go to compensate for the limitations of the scanner. For instance, heterogeneity features can be evaluated using a filtration-histogram based on the Laplacian of Gaussian (LoG) filter.

The limitations of the AI field are certainly the use of a small dataset, the use of only one scanner, the need for manual ROI, and the lack of external validation to confirm these results. Furthermore, for the reproducibility of the studies, it is important to adhere to the standardization of the radiomics features nomenclature and calculation according to the IBSI standard [43].

Another factor that may discriminate the presence of lipoma *vs.* WD-LPS may be the *MDM2* gene [44] from contrast-enhanced CT plus axial MRI T1W and T2FS one month prior to surgery.

Using the radiomic software ITK-SNAP [45], ROIs can be manually segmented on both CT and MRI, this tool complies with the IBSI standard.

Results showed no differences between the clinical distribution of training and testing, or between lipomas and WD-LPS distributions in the training set and external validation set. However, when these data are available, it is certainly possible to say that combining several imaging modalities suggested improved MDM2 amplification prediction abilities. The previously cited literature is shown in Table 3.

**Table 3:** State of the art about ST disease in radiomics

**Aim**	**Authors**	**Information database**	**Radiomic software**	**Imaging**
WD-LPS/ALT *vs.* lipoma	Cay et al. [24]	Histologic examination; MDM2 detection	IBEX	T1W TSE MRI
WD-LPS *vs.* lipoma	Vos et al. [37]	MDM2 detection	PREDICT	T1W and T2W MRI
LPS *vs.* lipoma	Malinauskaite et al. [26]	Histologic examination	Pyradiomics	T1W-SE MRI
LPS *vs.* lipoma	Leporq et al. [39]	Histologic examination; Multicentric imaging	MatLab	Contrast-enhanced T1W MRI
ALT *vs.* lipoma	Tang et al. [40]	Histologic examination	Research Portal V1.1	T1W and T2FS MRI
ALT *vs.* lipoma	Pressney et al. [41]	Histologic examination; fat heterogeneity information; MDM2 detection	TexRad	T1; PDW FSE MRI
WD-LPS *vs.* lipoma	Yang et al. [44]	Histologic examination; MDM2 detection	ITK-SNAP	Contrast-enhanced CT; T1W and T2FS MRI

SE: spin echo

The ability to diagnose STSs utilizing radiomics and AI technology has the potential to be more precise and effective. There are, however, a number of issues that must be resolved, including the requirement for sizable and varied datasets, the standardization of imaging methods, and the validation of AI models in clinical settings. AI combined with radiomics has the potential to enhance STSs risk classification, treatment planning, and monitoring. However, to fully exploit the potential of AI with radiomics in identifying and treating STSs, however, more study and collaboration between physicians and data scientists are required.

## Conclusions

In conclusion, it is evident how radiomics can offer decision support in the diagnosis of increasingly complex diseases. Diseases are different from each other, even if they belong to the same class. Much information obtained from images, which is neglected due to the limitations of the human eye and which may seem irrelevant, is decisive in the development of diagnoses and therapies. In particular, some studies have highlighted precisely the differences between automatic and radiological classification, calculating the metrics shown in Table 3.

If radiomics made it possible to distinguish the G of LPS, then it would be possible to diagnose the metastatic risk early in patients with a high-G neoplasm and consequently determine the subsequent course of treatment. Another advantage could be to avoid the incorrect grading of the lesion due to the non-optimal biopsy sampling. This would lead to a reduction in the risk of recurrence and greater possibilities in the therapeutic treatment of the tumor with positive and long-lasting outcomes. In the case of LPSs, or other malignant and aggressive diseases, which require invasive intervention, this technique would allow prognoses to be formulated in less time, without exposing the patient to further risks.

## Abbreviations

AI: artificial intelligence
ALT: atypical lipomatous tumor
AUC: area under the curve
CHT: chemotherapy
CT: computed tomography
DD-LPS: dedifferentiated liposarcoma
FSE: fast spin echo
G1: grade 1
IBSI: image biomarker standardization initiative
LPS: liposarcoma
M: myxoid
MDM2: murine double minute 2
ML: machine learning
M-LPS: myxoid liposarcoma
MPM-LPS: myxoid pleomorphic liposarcoma
MRI: magnetic resonance imaging
MSK: musculoskeletal
PDW: proton density-weighted
PM-LPS: pleomorphic liposarcoma
ROI: region of interest
STSs: soft tissue sarcomas
STTs: soft tissue tumors
T1W: T1-weighted
TSE: turbo spin echo
WD-LPS: well-differentiated liposarcoma
WHO: World Health Organization

## Declarations

### Copyright

© The Author(s) 2023.
